# Conditions under which college students cease learning

**DOI:** 10.3389/fpsyg.2023.1116853

**Published:** 2023-04-20

**Authors:** Jeffrey Coldren

**Affiliations:** Department of Psychological Sciences and Counseling, Youngstown State University, Youngstown, OH, United States

**Keywords:** learning cessation, feedback-based learning, machine learning, self-regulated learning, computational psychology

## Abstract

**Introduction:**

Effective learning involves the acquisition of information toward a goal and cessation upon reaching that goal. Whereas the process of learning acquisition is well understood, comparatively little is known about how or when learning ceases under naturalistic, open-ended learning conditions in which the criterion for performance is not specified. Ideally, learning should cease once there is no progress toward the goal, although this has never been directly tested in human learners. The present set of experiments explored the conditions under which college students stopped attempting to learn a series of inductive perceptual discrimination problems.

**Methods:**

Each problem varied by whether it was solvable and had a criterion for success. The first problem was solvable and involved a pre-determined criterion. The second problem was solvable, but with no criterion for ending the problem so that learners eventually achieved a highly accurate level of performance (overlearning). The third problem was unsolvable as the correct answer varied randomly across features. Measures included the number of trials attempted and the outcome of each problem.

**Results and Discussion:**

Results revealed that college students rarely ceased learning in the overlearning or unsolvable problems even though there was no possibility for further progress. Learning cessation increased only by manipulating time demands for completion or reducing the opportunity for reinforcement. These results suggest that human learners show laudable, but inefficient and unproductive, attempts to master problems they should cease.

## Conditions under which college students cease learning

Feedback-based learning is commonly used by humans (Fouragnan et al., 2015; Wang et al., 2018; Van Der Kooij et al., 2021) and computers (Kaelbling et al., 1886; Sutton and Barto, 1998) to acquire and revise information. The learner systematically generates and evaluates responses after being given feedback from the environment about the correctness of the response to meet a goal (e.g., Levine, 1975; Gholson, 1980; Murayama and Kitagami, 2014; Verburg et al., 2019). As such learning is necessary in any multidimensional problem to extract relevant information from competing stimulus demands, it forms the basis for induction (Nguyen-Xuan, 2020), concept formation (Bruner et al., 1956), categorization (Hughes and Thomas, 2021), and higher-order reasoning (Decker et al., 2016; Hespos et al., 2021).

To be successful, the learner must regulate between two states: Acquisition and cessation. Acquisition involves gathering enough information to solve a problem. Most research on learning has focused on this critical process. Equally important though, but less recognized by investigators, is the need for the learner to cease once adequate information has been accumulated (e.g., Shultz et al., 2012). The essential balance for learners to achieve is to amass enough information to solve the problem, but not so much that scarce time, energy, and limited cognitive resources are wasted through prolonged and unproductive learning attempts (Lucas et al., 2015). Simply put, the learner must determine when they have learned enough to solve the problem, or when are they wasting time on a problem that they either already know or will never learn.

### Experimenter-controlled versus learner-controlled conditions

The decision when to cease learning is obvious when an external and explicit criterion for mastery has been imposed upon the learner as is typical in most experimenter-controlled, laboratory-based studies (e.g., Skinner, 1938). Once performance reaches the predefined level of mastery, learning is terminated by the experimenter (Pitts and Hoerger, 2021). As a result, most investigators have given little consideration as to when the learner would cease if left unconstrained. One interesting exception is Nevin’s (Craig et al., 2014) observation that a behavioral response may gather momentum to continue beyond the criterion, similar to physical forces operating on an object. Although responding cannot continue *ad infinitum* due to fatigue by the learner or satiation to the reinforcer, just how long it continues has not been a matter of concern due to the strong control exercised by the experimenter.

Learning under natural conditions poses a unique set of challenges about when to cease acquisition (e.g., Dunlosky and Rawson, 2019). First, not all problems in the natural world have defined solutions (if at all); and second, not all solutions specify a predetermined level of mastery to be achieved (Ferguson, 1989). The learner must decide when a satisfactory level of performance has been attained and therefore when to stop. Consider, for example, when to stop interviewing candidates as potential employees for an organization. There are an extensive number of applicants, but a finite amount of time for the interviews. After doing the initial screening, interviews are conducted and there is some assessment of suitability of the candidate. How long would be appropriate to continue interviews before making a choice to hire to end the search? In another example, a driver is searching for an empty parking space on a crowded street. There are several available spaces that vary in distance from destination and size of the space. How long should the driver search until making a choice? Both these examples are variations on the classic ‘secretary’ problem in mathematics (Ferguson, 1989). These examples of natural learning situations bear some similarity to educational techniques such as problem-based and project-based learning in which the learner must autonomously determine the end state (Wood, 2003; Allen et al., 2011; Kokotsaki et al., 2016; Bernardo et al., 2019). In these open-ended conditions, being an efficient and autonomous learner involves deciding the optimal time to stop making attempts given an uncertain outcome (Chow et al., 1971; Hill, 2009).

There is uncertainly though whether humans can adequately judge their own learning to determine when to stop attempts. Atkinson (1972), for example, was dubious of whether learners could make decisions about their progress, leading him to advocate an instructor-driven rather than a student-driven approach to education. This doubt is increased by recent reviews that humans have a great deal of difficulty learning from failure (Eskreis-Winkler and Fishbach, 2019, 2022). As schools and universities increasingly adopt learner-centric methods of instruction rather than instructor-driven (Herrington et al., 2014; Scheiter, 2020), these criticisms do not bode well for successful autonomous human learning. Unfortunately, there is little theoretical and empirical basis to support any conclusion about the effectiveness of humans to cease their own learning.

### Explanations of cessation in human learning

The self-regulation of learning model (SRL) model explicitly considers the metacognitive control of learning (Zimmerman and Schunk, 2001; Dunlosky et al., 2005), but is vague on the process of cessation. This model identifies that learners set goals, evaluate progress toward those goals, and revise behavior toward the goal (Butler and Winne, 1995; Winne, 2001). As such, the SRL model predicts that learning *should* stop once the goals are met, but it does not specify the actual conditions under which learners do stop learning (i.e., Eskreis-Winkler and Fishbach, 2019), nor does it account for cases in which learning continues beyond what is effective to solve the problem (Lucas et al., 2015). This may be due to the reliance of the SRL model upon self-report questionnaires that may not reflect actual learning performance in natural learning situations (Zhou and Winne, 2012; Ventura et al., 2013; Friedman and Gustavson, 2022).

The SRL model, however, has generated several explanations of how much time a learner should allocate in a related task such as studying. The discrepancy reduction model specified that humans make judgments to allocate study time toward information that is most discrepant from some internal criterion (Nelson and Dunlosky, 1991; Dunlosky and Hertzog, 1998; Dunlosky et al., 2013). A limitation of this explanation though is that it cannot explain why individuals who allocate a great deal of time to very difficult items have such little gain, called the labor-in-vain effect (Nelson and Leonesio, 1988). To explain why college students invested more time studying difficult items, despite having less success and lower confidence, the diminishing criterion model proposed that goals are adjusted downward over time (Ackerman, 2014; Undorf and Ackerman, 2017). The third model, the region of proximal learning framework, held that individuals study those items that are slightly beyond their current level of knowledge (Metcalfe, 2002, 2009; Metcalfe and Kornell, 2003). Further, learners judge their rate of learning as the basis for determining when to stop (Metcalfe and Kornell, 2005). When the rate of learning proceeded quickly, learners continued to study. When the judgment of the rate of learning came to a standstill, learners stopped.

Although these models have been designed to explain study time allocation rather than feedback-based learning, they raise the important point that continuation of learning should be related to the amount of progress. Specifically, learning should end upon reaching a point of diminishing returns. This is a key tenet of the theory of educational productivity that learning should be economical (Walberg, 1982). Two experiments highlight this point. Murayama et al. (2016) allowed college students the explicit choice as to when to quit studying material. Results revealed that participants who stopped studying early recalled less information. The decision to quit early may be due to the judgment that further studying would yield little benefit given the effort involved (e.g., Nelson and Leonesio, 1988). Another experiment by Payne and Duggan (2011) found that learners sought to maximize payoff and quit tasks that afford few opportunities for success (e.g., Anderson, 1990). They asked college undergraduates to solve a series of problems with the understanding that some would be unsolvable. Learners spent more trials attempting problems that were solvable and contained more problem states, whereas they were quicker to quit unsolvable problems. Both these experiments suggest the decision to cease learning is based on a judgment of potential outcome; when the likelihood of future success is low, the optimal decision is to quit. This conclusion, however, is contradicted by the puzzling labor-in-vain effect that humans may spend an inordinate amount of effort on tasks that yield little payoff (Nelson and Leonesio, 1988; Lucas et al., 2015).

### Explanations of cessation in machine learning

Like humans, when to cease learning is critical for successful machine learning and artificial intelligence applications (Vlachos, 2008; Ishibashi and Hino, 2020; Li et al., 2020). A desirable quality of any autonomous organism is the ability to function independently from external intervention or supervision which involves self-regulation of information acquisition and cessation (e.g., Shultz et al., 2012). Therefore, borrowing insights from machine learning agents may inform the study of human learning cessation under open-ended conditions (Shultz, 2003; Sun, 2008; Mills et al., 2011).

To explore learning cessation, Shultz et al. (2012) used a computational simulation approach by examining the parameters under which a neural net model ceased learning. The goal of the model was to classify perceptual values into one of four possible outcomes. As this was a supervised model (e.g., Mohri et al., 2018), the difference between the current accuracy of the classifications and the correct values produced a state of error. The model evaluated learning progress in relation to the degree of error reduction. Simulations revealed that learning continued if there was a reduction in error between the present state and desired outcome; Learning ceased once errors were reduced and maintained at a stable level. Moreover, learning ceased faster when only half of the stimulus patterns were correctly identified (e.g., 50% learnability) rather than when all the patterns were correctly identified (100% learnability). With less consistent information available to learn, error reduction stalled at a higher level, thus causing the model to end faster than when more information available was available. Whether human learners also cease making attempts upon reaching a stable and low rate of error forms the rationale of the present set of experiments.

### Purpose of the experiments

In summary, there is general agreement among models of human and machine learning that the decision to cease learning should be based upon an evaluation of the current state of progress, although this has never been directly tested. Ideally, the learner would judge the adequacy of current progress against the costs of continuing (Kurzban et al., 2013). As long as progress is made toward the goal, the learner should continue to attempt a problem (Nelson and Narens, 1990). When there is little additional information to be gained, such when a task is either impossible or has been mastered, it is optimal to stop (Ariel et al., 2009). There is no further information to be gained once learning reaches a high state of accuracy or a sustained level of error. Continued attempts would waste time or cognitive resources and limit further learning opportunities (Buchanan, 1991; Kurzban et al., 2013).

Given the limited understanding of when or if humans cease learning, and the importance of this ability for human autonomous learning in naturalistic conditions, the specific purpose of this project was to measure the number of trials attempted by college students before they cease learning under various experimental conditions and levels of success. Specifically, it was tested whether learning ends once there is no further gain in progress.

### General method and procedure

**Figure 1:**
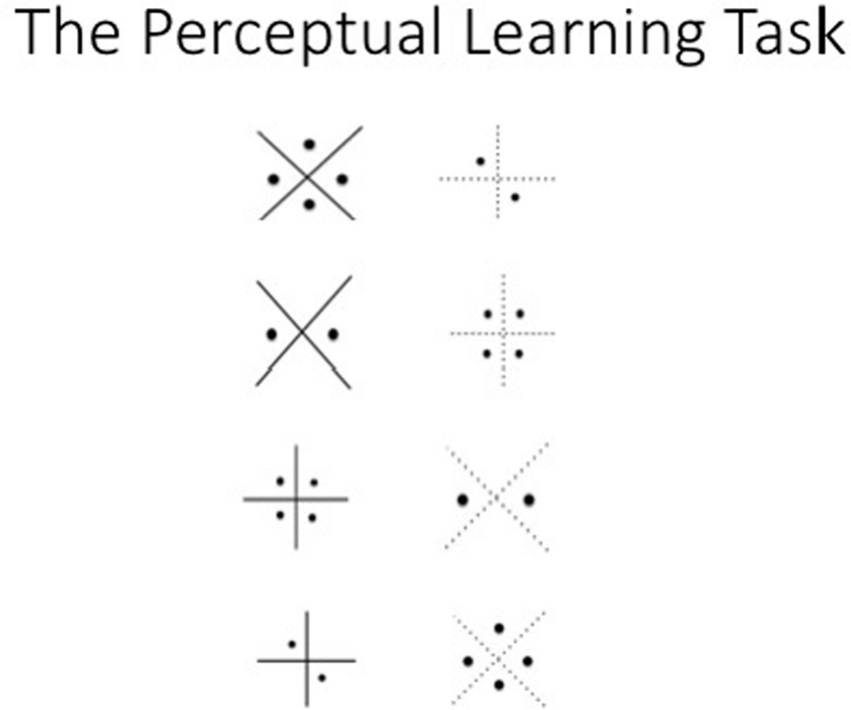
Stimuli Presented in the Perceptual Learning Task.

A feedback-based learning task was created that allowed the control of problem solvability and solution criteria. Participants solved three consecutive perceptual discrimination problems presented on a computer programmed using PsychoPy (Peirce, 2007, 2008). Each problem consisted of a pair of abstract geometric figures with four co-varying perceptual dimensions (see Figure 1 for an example of the stimulus pairs presented over the first four trials). The experimental features were counterbalanced across blocks of eight trials so that all attributes appeared in every position. This was an inductive learning task in which the correct choice was initially unknown to the participant; they were to select the one feature that was consistently rewarded over trials. Participants pressed the left or right keyboard arrow keys to indicate their choice. Feedback about the correctness of the choice (correct or not correct) appeared on the screen after every response. Participants were given verbal (at the beginning of every problem) and written (at the bottom of every trial screen) instructions informing them of the option to quit the current problem.

Two practice problems were given to acquaint participants with the procedure, followed by three experimental problems. Each experimental problem varied by whether a solution was possible and whether an external criterion for success was imposed upon the learner. Each problem explained below contained a maximum of 64 trials.

One problem was solvable meaning that one feature was designated as correct that was to be identified by the learner. Further, an external learning criterion defined as eight consecutive correct responses was imposed upon the learner. This problem served as the control to assess how participants solved the problem under typical mastery-learning conditions.

The second problem also contained a defined correct feature thus making it solvable, but it did not contain an externally imposed criterion for stopping. Without any constraint to end learning, participants could make as many attempts as they wished until reaching the maximum of 64 trials. As maintained by hypothesis-testing theory, adult learners typically do not reject the correct hypothesis once selected (Restle, 1962); therefore, it is possible that learners could attain high success rates if they did not end the problem, leading to overlearning.

The third problem was unsolvable as no individual feature was consistently rewarded and only 50% of the trials contained any reinforcement regardless of the response. This problem did also did not contain a learning criterion so the participant could continue unabated until reaching the maximum of 64 trials. It was expected that learners would likely attain a moderate and constant success rate as the level of success hovered around chance levels performance over trials (e.g., 50%).

Outcome measures were the number of trials attempted on each problem and the problem outcome (i.e., whether the participant solved the problem, quit the problem, or attempted the maximum number of trials). Over the course of five separate experiments, experimental demands upon the learner were manipulated to determine the conditions under which learners were mostly likely to cease the three types of problems.

## Experiment 1

The first experiment tested whether learning ceased when participants reached either a consistently high level of success (when no more learning is possible) or when learning stalls around chance-level performance (when the problem will never be mastered). The first case would be reflected in the overlearning problem, and the second would be represented in the unsolvable problem. In either case, it was expected that most participants would quit the problem before reaching the maximum number of trials. These predictions were initially tested under ideal performance conditions as participants were instructed to attain as many correct responses in a row as possible.

### Participants, methods, and procedure

Students from the department participant pool volunteered to satisfy research requirements. The university IRB approved the project. Participants signed a consent form that assured their anonymity, stated their right to withdraw, and explained the option to choose an alternative assignment to complete the research requirement.

The sample contained 67 college students (69% females; 33% non-Caucasian) with a mean age of 22.22 years. The mean number of credit hours completed was 40.95. The mean college GPA was 3.21 (out of 4.00).

The procedure for the learning task described in the general method was followed. In this *Maximum Effort Condition,* participants were instructed to attain as many correct responses in a row *as possible*.

### Results and discussion

The analysis strategy described below was followed for all experiments in this report. Analyzes were conducted using R Studio (2020). All data and code are available on the Open Science Framework. 

#### Trials over problems

The number of trials attempted differed across the solvable (*M* = 12.19), overlearning (*M* = 51.72), and unsolvable problems (*M* = 48.48) as revealed by significant differences in a one-way related-groups ANOVA [*F*(2,132) = 168.65, *p* < 0.05]. All LSD pair-wise comparisons were significant at the 0.05 level. Participants attempted the fewest number of trials in the solvable problem and attempted more trials in the overlearning and unsolvable problems.

#### Problem outcome

Inspection of the number of trials attempted for each problem does not give any insight into the outcome, so the next analysis examined the percentage of participants who attained each outcome. The outcome for each problem was examined in separate analyzes.

**Figure 2:**
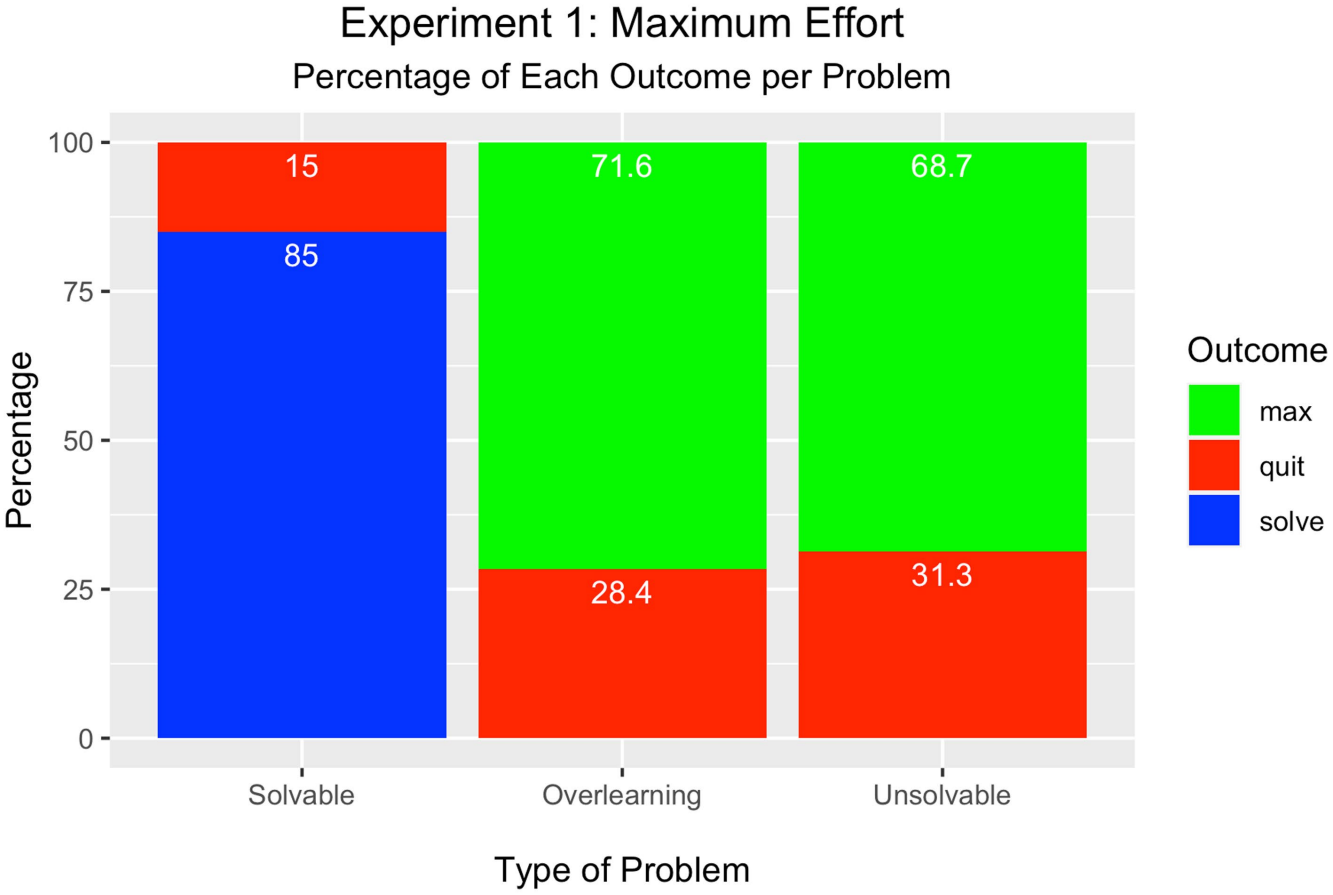
Experiment 1 – Maximum Effort Condition.

For the solvable problem, one of three outcomes is possible. Participants may either solve the problem, quit the problem, or attempt the maximum number of trials. As displayed in Figure 2, most learners attained success on the solvable problem (85%) and few quit this problem (15%). None attempted the maximum number of trials. These percentages were tested in a Chi-square one-way test of significance and found to be significantly different from each other [*Χ*^2^(2) = 123.5, *p* < 0.001].

There are only two outcomes possible for the overlearning and unsolvable problems (quit or attempt the maximum number of trials) as the criterion for success was removed to prevent problem solution. In the overlearning problem, most participants attempted the maximum number of trials (72%) rather than quit the problem (28%); The percentages for these outcomes were significantly different from each other [*Χ*^2^(1) = 19.36, *p* < 0.001].

In the unsolvable problem, the number of participants who attempted the maximum number of trials was significantly greater (69%) than the percentage who quit (31%) [*Χ*^2^(1) = 14.44, *p* < 0.001].

#### Summary

Most participants had success on the solvable problem and rarely quit either the overlearning or unsolvable problems even though no success was possible. Instead, most learners persisted until reaching the limits of the task rather than quit. This performance runs counter to the prediction that learning should cease when no progress is made toward successful solution. This outcome, however, is not entirely surprising as learners were explicitly instructed to achieve their best performance so accumulating as many trials as possible is a viable strategy as there is no cost relative to potential gain. Given this performance as a baseline, the following experiments manipulated conditions to observe whether there was an increase in learning cessation.

## Experiment 2

The results of the first experiment reflect ideal learning performance; participants attempted many trials to attain their best performance. The purpose of the second experiment was to determine if cessation increased when participants engaged in self-evaluation of their learning progress as predicted by the SRL model (e.g., Winne, 2001). If participants self-reflect to monitor their progress, they may be more likely to cease learning upon reaching performance levels that are either highly accurate or consistently inaccurate. As learners would hold a lower criterion for performance other than the ideal, it is expected they would be more likely to cease further attempts at learning once they realize their lack of progress. To test this prediction, in this *Reflection Condition,* participants were told to attain as many correct responses in a row *until they felt confident they solved the problem*.

### Participants, methods, and procedure

This sample contained 39 college students (49% females; 36% non-Caucasian) with a mean age of 22.97 years. The mean number of credit hours completed was 39.79, and the mean GPA was 3.24 (out of 4.0).

The method of the second experiment was almost identical to the first with the exception that participants were told to attain as many correct responses as possible in a row until they felt confident they had solved the problem. The analytic strategy was also the same as the first experiment.

### Results and discussion

#### Trials over problems

There were significant differences in the number of trials attained across the solvable (*M* = 23.31), overlearning (*M* = 47.72), and unsolvable problems (*M* = 55.13) [*F*(2, 76) = 48.65, *p* < 0.05]. All LSD pair-wise comparisons were significant at the 0.05 level.

#### Problem outcome

**Figure 3:**
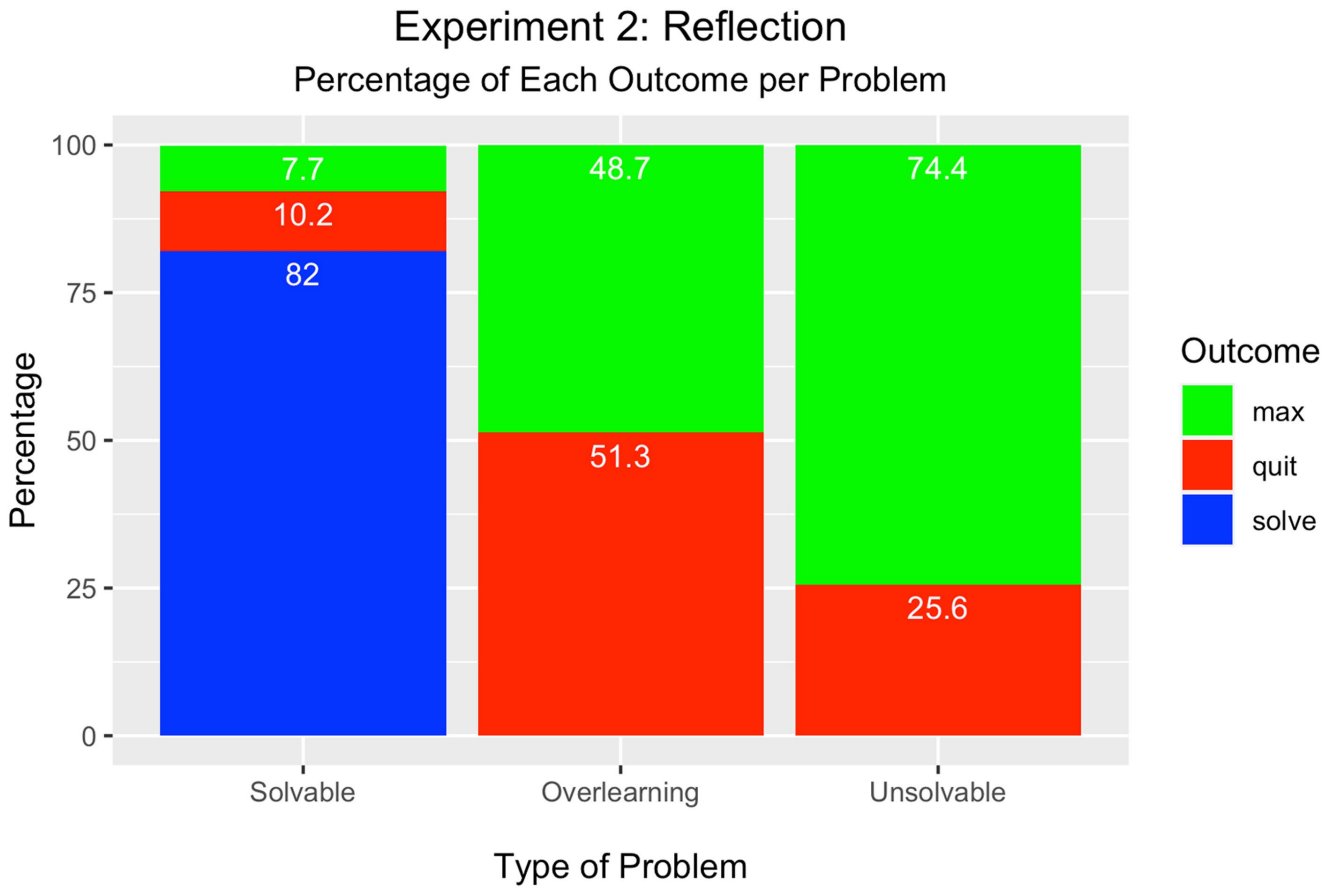
Experiment 2 – Reflective Effort Condition.

As displayed in Figure 3, most students solved the solvable problem (82%), whereas the minority either quit (10%) or attempted the maximum number of trials (8%). These percentages were tested in a Chi-square test of significance and found to be significantly different from each other [*Χ*^2^(2) = 106.60, *p* < 0.001].

In the overlearning problem, however, there was no difference between the percentage of participants who attained the maximum number of trials (49%) and those who quit (51%) [*Χ*^2^(1) = 0.04, *p* > 0.05].

There was a significant difference between the percentage of participants who attempted the maximum number of trials in the unsolvable problem (74%) compared to the percentage who quit (26%) [*Χ*^2^(1) = 23.04, *p* < 0.001].

#### Summary

The most interesting finding from this experiment was that an approximately equal percentage of learners either quit the overlearning problem or attempted the maximum number of trials. This suggests that the instructions to self-monitor had the desired impact to reduce the number of trials attempted on the overlearning problem. The instruction to engage in self-reflection did not have any impact upon solution of the unsolvable problem. Most learners did not quit, but instead continued until reaching the maximum number of trials. The decision to continue to attempt either the overlearning and unsolvable conditions is inefficient and unproductive as there was little benefit to be gained because learning was either highly accurate or hovered around chance-level performance with no chance of improvement.

## Experiment 3

Given the unexpected finding that most participants did not quit the unsolvable problem, it was conjectured that a history of efficacious learning experience from the prior solvable problem may have biased participants’ estimation for success on later problems. The experience of achievement in the first problem may have set the expectancy that later problems will also be solvable (Eisenberger, 1992). The purpose of the third experiment, therefore, was to test whether the order of problems increased expectation of success, and therefore, influenced the decision to quit or continue in later problems. The possibility of a carryover of learning experience across problems was tested in this *Reverse Order Condition* by giving the unsolvable problem at outset. Following evidence that performance is impaired following unsolvable problems (Frankel and Snyder, 1978; Mikulincer, 1988), it was predicted that if later problems would be differentially impacted by the initial experience of failure, quitting should be higher in the later problems. If the problems are treated independently by participants and prior experience has no effect, the rates of quitting for each problem will remain the same as previous experiments.

### Participants, methods, and procedure

This sample contained 41 students (59% females; 34% non-Caucasian) with a mean age of 19.37 years. The mean credit hours completed was 28.9, and the mean GPA was 3.44 (out of 4.0).

The type of problems was the same as the previous experiments except their order was reversed so that the unsolvable problem was presented first, followed by the overlearning problem, and then by the solvable problem.

### Results and discussion

#### Trials over problems

The number of trials attempted across the unsolvable (*M* = 58.00), overlearning (*M* = 39.44), and solvable (*M* = 17.31) problems were all significantly different [*F*(2, 80) = 67.94, *p* < 0.05]. Even though the order of the problems was reversed compared to the prior experiments, the solvable problem was solved easily (even though it was last in the sequence) whereas participants spent more trials attempting to solve the overlearning and unsolvable problems.

#### Problem outcome

**Figure 4:**
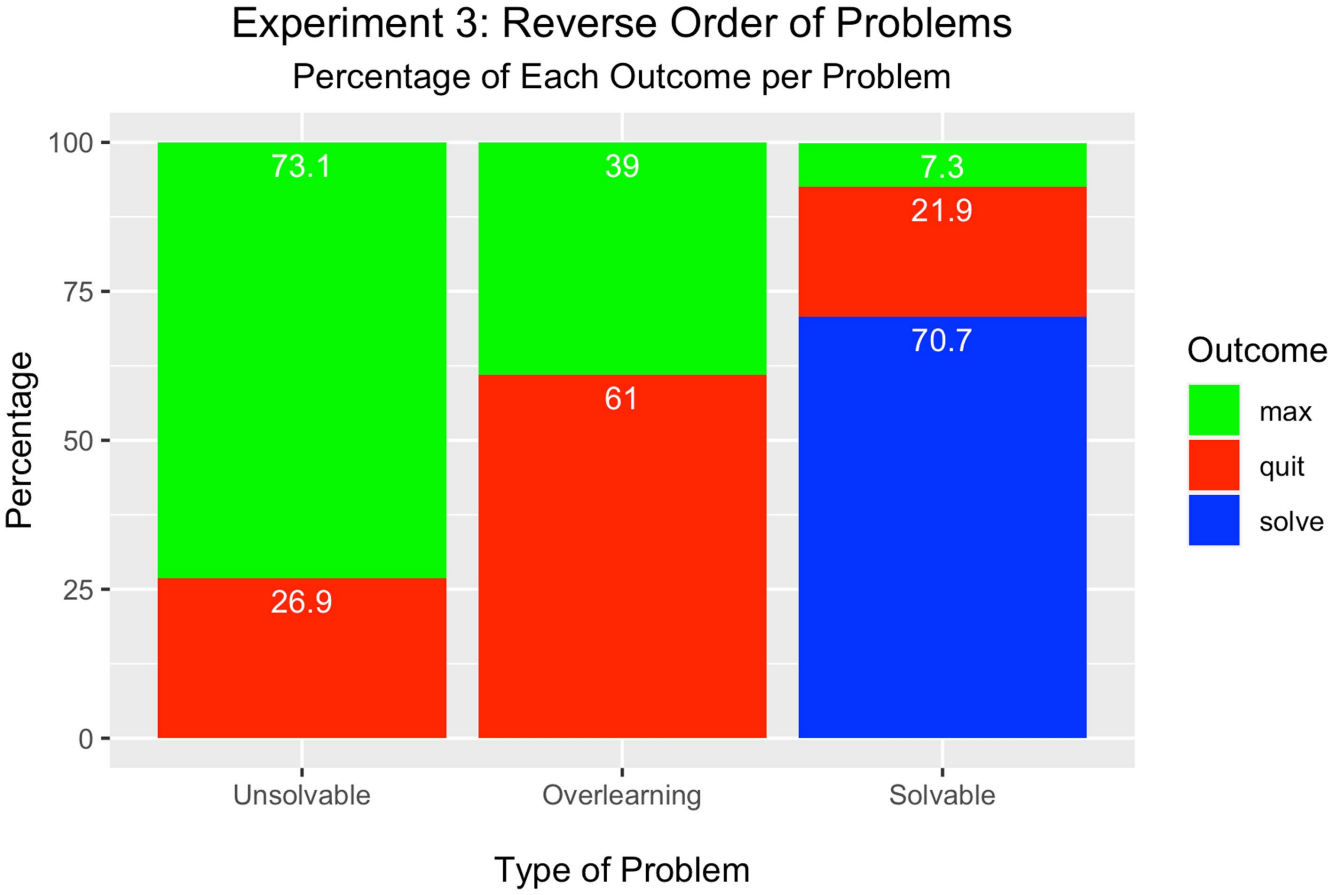
Experiment 3 – Reverse Order Condition.

As shown in Figure 4, most participants attempted the maximum number of trials (73%) in the unsolvable condition compared to the percentage who quit (27%); this difference was significantly different [*Χ*^2^(1) = 21.16, *p* < 0.001]. Such performance was similar to participants in the unsolvable condition in Experiment 2, even though this condition was now presented first in the sequence.

In the overlearning problem, more participants quit the problem (61%) compared to those who attained the maximum number of trials (39%) [*Χ*^2^(1) = 4.84, *p* < 0.05]. This is the first finding thus far that quitting was the most frequent solution to the overlearning problem.

Like in Experiments 1 and 2, most students solved the solvable problem (71%), which now appeared last in the sequence. The minority either quit the problem (22%) or attained the maximum number of trials (7%). These percentages differed significantly from each other [*Χ*^2^(2) = 67.19, *p* < 0.001].

#### Summary

Leading with a high rate of failure from the unsolvable problem increased the tendency to quit a problem, but only in the overlearning condition. There was no impact upon quitting in the unsolvable problem; the participants were still remarkably tenacious in spite of the fact that they would never solve the problem. It is also important to point out that performance did not change in the solvable problem even though it occurred last in the sequence. Thus, it may be concluded that prior successful experience did not have any bearing upon cessation, particularly when the initial problem was not capable of being solved.

## Experiment 4

The experience of prior failure made participants more likely to cease the overlearning problem, but not the unsolvable problem. Why was quitting uncommon in the unsolvable condition given only half (50%) of the trials had feedback and no feature was consistently rewarded? Recall from the predictions of the neural net model that learning ceased faster under conditions of less learnability (i.e., fewer available reinforced stimuli). In contrast to the neural net model, 50% learnability may still be too rich in the unsolvable problem to entice human participants to quit. The neural net model tested even lower learnability ratios with the effect that cessation became more likely with rates of available reinforcement less than 50%. Therefore, the purpose of this experiment was to test whether reducing feedback for the unsolvable problem from 50 to 25% with the prediction that human learners would be more likely to cease learning, especially in the unsolvable problem in this *Lower Reinforcement in Unsolvable Problem Condition.*

### Participants, methods, and procedure

This sample contained 32 students (66% females; 16% non-Caucasian) with a mean age of 20.41 years. The mean credit hours completed was 50.5, and the mean GPA was 2.92 (out of 4.0).

The procedure was the same as the reversed order in Experiment 3, but with a reduction in the number of reinforced trials in the unsolvable problem from 50 to 25%. This was accomplished by providing reinforcement on only two trials in a block of eight. Of the trials that were reinforced, feedback was not associated with any consistent feature.

### Results and discussion

#### Trials over problems

The number of trials attained across the unsolvable problems (*M* = 50.10), overlearning (*M* = 37.28), and solvable (*M* = 18.03) problems were all significantly different [*F*(2, 62) = 27.55, *p* < 0.05].

#### Problem outcome

**Figure 5:**
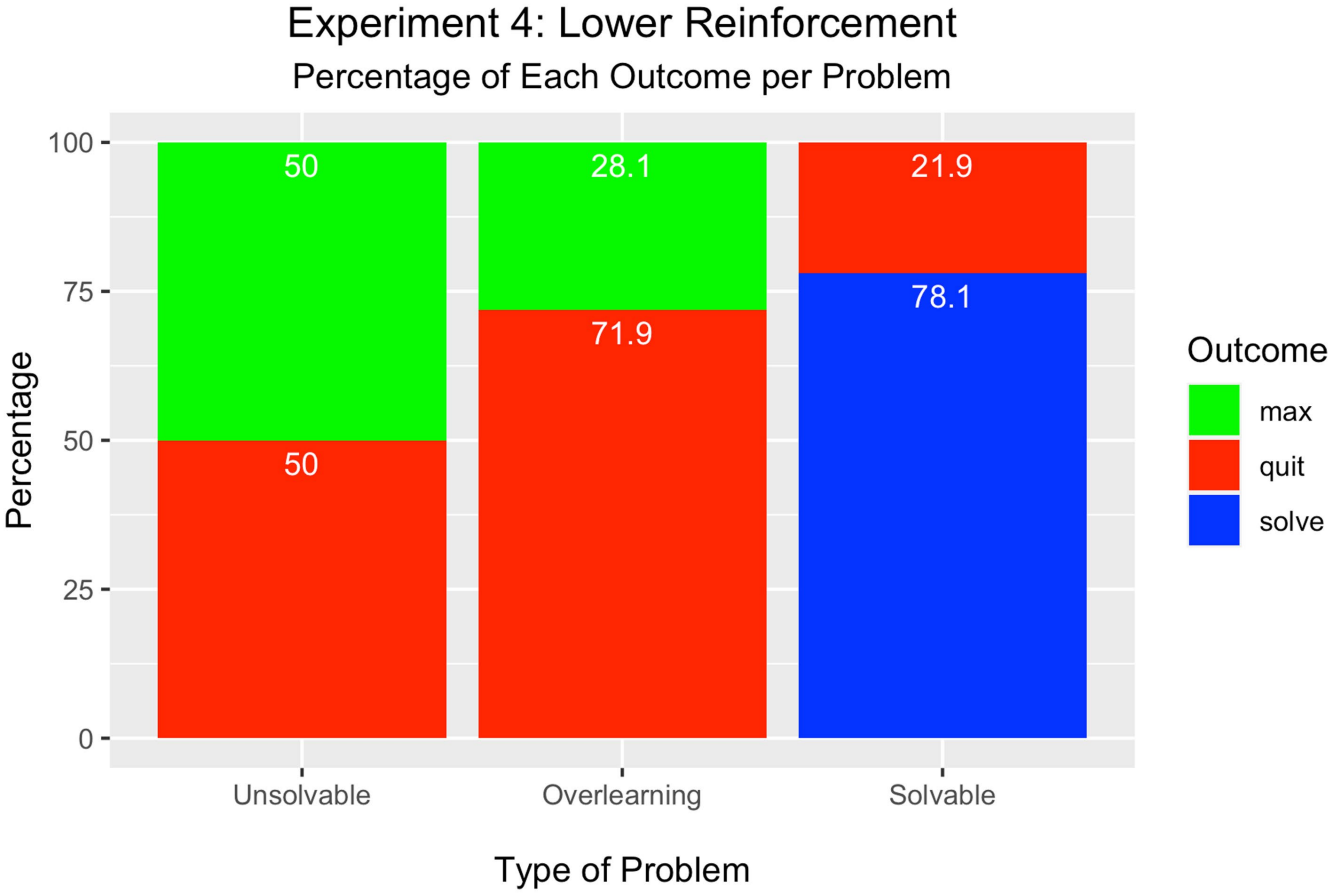
Experiment 4 – Lower Reinforcement Condition.

As depicted in Figure 5, an equal percentage of learners either quit (50%) or attempted the maximum number of trials in the unsolvable problem (50%) [*Χ*^2^(1) = 0.00, *p* = ns]. This is the highest level of quitting observed in this series of experiments.

As in Experiment 3, however, a difference was observed in the overlearning problem such that more participants quit the problem (72%) compared to those who attained the maximum number of trials (28%) [*Χ*^2^(1) = 19.36, *p* < 0.001].

Further, most learners solved the solvable problem (78%), whereas the minority quit the problem (22%) [*Χ*^2^(2) = 97.00, *p* < 0.001]. None attained the maximum number of trials.

#### Summary

Changing the learning experience by decreasing the availability of reinforcement had the effect of increasing cessation in the unsolvable problem; the level of cessation was equal to the number of learners who attempted the maximum number of trials. These results are in line with predictions of the neural net model that learning ceases faster when the problem contains less reinforced information.

## Experiment 5

The results of the previous four experiments suggest remarkably consistent attempts at learning under conditions in which cessation should be the efficacious outcome given the futility of the problem. The last experiment tested whether having to manage increased time demands would induce participants to engage in self-regulation of learning, and therefore cease learning particularly in the unsolvable problem. To do this, in this *Timed Condition*, the amount of time allowed to complete the three problems was decreased, which presumably would increase the time pressure and thereby raise cessation across all problems.

### Participants, methods, and procedure

This sample contained 41 students (76% females; 20% non-Caucasian) with a mean age of 19.59 years. The mean credit hours completed was 29.75, and the mean GPA was 3.49 (out of 4.0).

Following the procedure and order used in experiments three and four, participants were notified in the present experiment that they had 5 min at the outset of the experimental session testing to complete all three problems. The time remaining was announced by an experimenter at 4, 3, 2, 1 min, 30 s, and 10 s.

### Results and discussion

#### Trials over problems

The number of trials attempted across the unsolvable problems (*M* = 46.76; *n* = 41), overlearning (*M* = 30.27; *n* = 40), and solvable (*M* = 13.36; *n* = 28) problems were all significantly different [*F*(2, 66) = 25.77, *p* < 0.05]. Notice that the number of participants who completed each problem decreased because of the increased time demands. In other words, if participants ran out of time during the second problem, for instance, they would not be available to attempt the last problem.

#### Problem outcome

**Figure 6:**
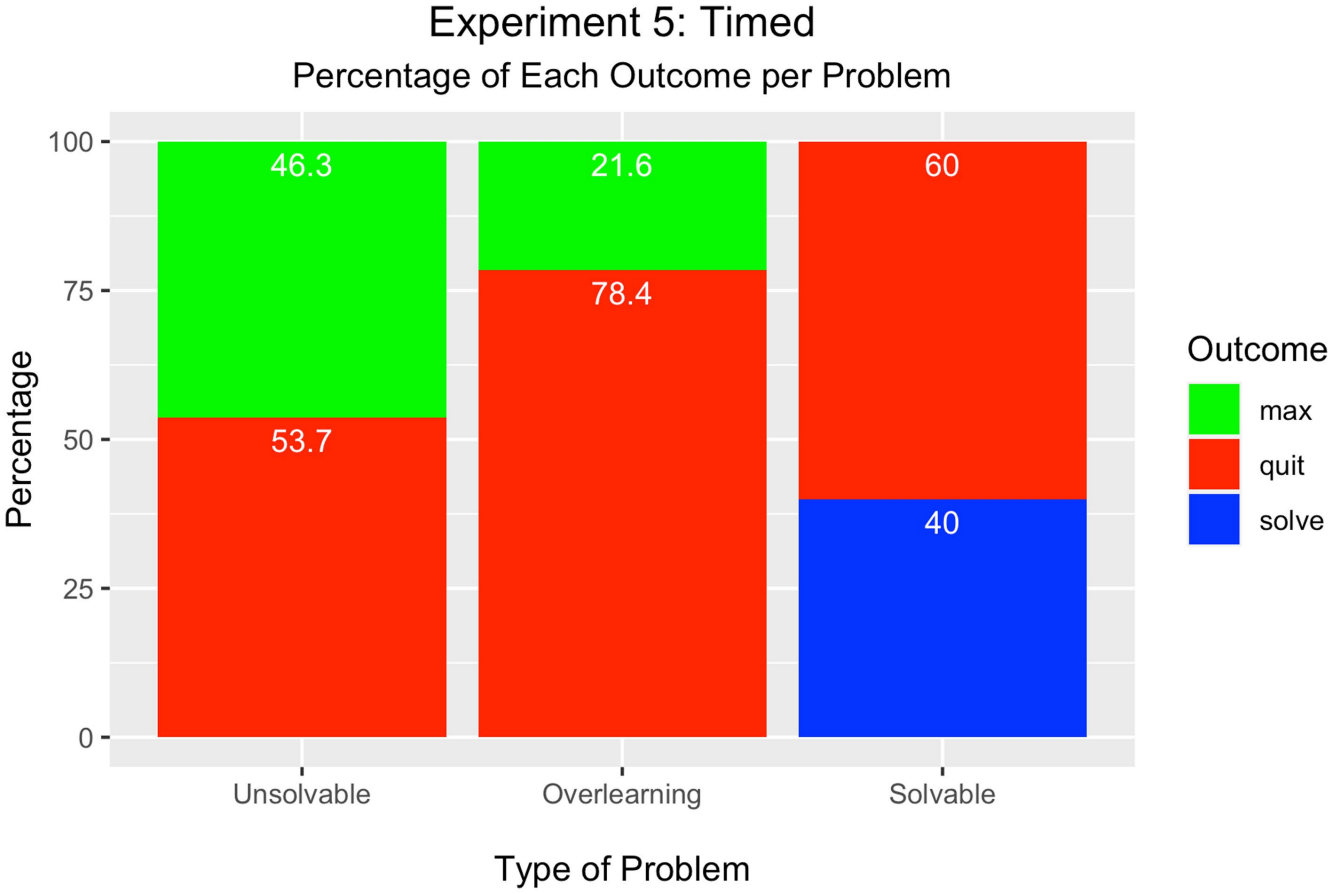
Experiment 5 – Timed Completion Condition.

As displayed in Figure 6, for the first time across the experiments, quitting was the most frequent outcome in all problems.

There was a slightly higher percentage of learners who quit the unsolvable problem (54%), compared to the percentage who attained the maximum number of trials (46%) although the difference was not significant [*Χ*^2^(1) = 0.64, > 0.05].

In the overlearning problem, there was a significant difference between the percentage of learners who quit the problem (78%) compared to those who attained the maximum number of trials (22%) (7%) [*Χ*^2^(1) = 31.36, *p* < 0.05].

In the solvable problem, most learners quit (60%) rather than solve the problem (40%); there were no participants who attempted the maximum number of trials. The percentages were significantly different from each other [*Χ*^2^(2) = 46.66, *p* < 0.001].

#### Summary

Adding time pressure to complete the learning tasks had the effect of making learning cessation the most common outcome across the tasks. Informing participants of the amount of time they had to allocate to the three problems presumably caused them to self-reflect and manage cognitive resources judiciously. This planning had the effect that more participants ended the task presumably because additional learning would come with more cost than benefit.

## General discussion

These five experiments provide consistent evidence that college students display a high number of attempts to solve problems, including those that yield little return from effort. Under most situations, this would be laudable; but it is inefficient and unproductive when involving overlearning or unsolvable problems. Cessation of these problems would be the wise course of action as precious time could be invested in attempting tasks that offer no learning benefit.

In the first experiment, participants attempted solvable, overlearning, and unsolvable problems with the instructions to achieve optimal performance. Most learners continued until they achieved success on the solvable problem; Most also continued to attempt the overlearning or unsolvable problems. Unlike the solvable problem, the overlearning and unsolvable problems did not impose any criterion for performance. The decision whether to continue or quit was left to the learner. When learners were to reflect upon their solution confidence (Experiment 2) to engage in self-regulated learning, most still attempted the maximum number of trials in the unsolvable problem. That few participants quit this problem is counterintuitive because there was little progress to be gained by continuing. The overlearning problem encouraged high levels of accuracy, therefore no further increase in learning performance was possible. Further, no progress toward learning could be made in the unsolvable problem because it was designed that no solution could be attained. Unlike the first experiment which directed participants to attain their best performance, the second experiment explicitly asked participants to engage in self-reflection which would presumably encourage cessation as the futility became evident. The third experiment found that reversing the order of the problems had little effect on increasing quitting in the overlearning and unsolvable problems suggesting that attempts were not due to prior successful experience or expectations. Reducing the amount of feedback in the unsolvable problem (Experiment 4) and increasing the time pressure to complete the problems (Experiment 5) did have the effect that more participants ceased learning compared to the other experiments, but many persisted in vain to solve the problem. It was particularly surprising that across all problems and experiments, learning in the unsolvable problem was the most resistant to cessation. The rate of success never rose above 50% (i.e., chance levels), suggesting that college students attempted these learning tasks without regard for their lack of progress.

### Explanations and models

The findings that many college students continued attempts at learning instead of ceasing the overlearning and unsolvable problems do not fit within existing explanations of self-regulated learning, such as the discrepancy reduction model (e.g., Nelson and Dunlosky, 1991), the diminishing criterion model (e.g., Ackerman, 2014) or the region of proximal learning model (e.g., Metcalfe and Kornell, 2005). One obvious reason for the lack of fit is that these models have been developed to explain study time allocation rather that feedback-based learning tasks. However, even considering this difference, the present results still do not conform to the central tenet of these models that learning should occur with some awareness of its effectiveness. Nevertheless, one point of agreement is the similarity between the extended attempts at learning in the present experiments to the labor-in-vain effect (Nelson and Leonesio, 1988), a fact that these models also cannot explain. Future revisions of self-regulation of learning models should attempt to incorporate the observation that some learners fail to stop making attempts given their lack of progress. These prolonged attempts at solving problems does appear to confirm the observation from Neville regarding behavioral momentum of a response past the criterion (Craig et al., 2014). In this phenomenon, the learning response was equated to the physical property of inertia on a moving object, which continued unabated unless another force acted upon it.

The present results also do not fit findings from machine learning algorithms that cease acquisition once there is no longer a reduction in error as revealed by the simulations by Shultz et al. (2012). Unlike machine agents, human learners amassed many trials as they failed to cease once they attained either mastery (overlearning problem) or reaching futility (unsolvable problem). Therefore, human learners appear to be insensitive to the reduction of the error rate, in contrast to their machine learning counterparts.

When viewed in terms of cost versus benefit, the findings from this project suggest some illogical or irrational behavior by not stopping once it was clear there was no progress toward the goal (Tversky and Kahneman, 1974; Kahneman, 2011). For example, an economically rational agent would have quit rather than continue their commitment (Keil et al., 2000; Bazerman and Moore, 2009). Whether we can say that this lack of cessation meets the level of irrationality is difficult to ascertain (Ackerman et al., 2020), but it can be concluded that repeated vain-glorious attempts are inefficient by squandering limited cognitive resources (Murayama et al., 2016) as well as failing to take advantage of more fruitful learning opportunities (Buchanan, 1991).

Given the unexpected observation that learners generally failed to quit nonproductive problems, the obvious but heretofore unaddressed question is why. Several psychological explanations may be offered to account for why participants engaged in dozens of trials without quitting with no change in the probability of success. The status-quo bias, identified by Samuelson and Zeckhauser (1988), refers to maintaining one’s current decision. In the context of the present project, the status quo refers to continuing the learning task and failing to quit long after it becomes informative. Maintaining the status-quo may avoid transition costs associated with making any decision change even if the outcome is unknown (Samuelson and Zeckhauser, 1988). Further, as humans are motivated to avoid losses, the perception that a change may result in even worse performance may make it desirable to stay with the present course of action (Thaler, 1980; Kahneman and Tversky, 1984). Learners may decide to maintain their psychological commitment by continuing to attempt learning trials. The larger the past investment in a decision, even if it is faulty, the more likely it will be continued as it represents a sunk cost (Thaler, 1980) or an attempt to avoid regret for past decisions (Kahneman and Tversky, 1982). In other words, learners may double down on past erroneous responses rather than give them up. Finally, there is also the possibility that learners may not be sensitive to the feedback for their performance. For instance, the metacognitive illusion holds that learners feel they are doing better than they actually are (Cervin-Ellqvist et al., 2020). Or, there are recent findings that humans have difficulty learning from failures or past errors (Eskreis-Winkler and Fishbach, 2019, 2022), even though there is educational benefit to be gained from making and correcting errors (Metcalfe, 2017). The failure to learn from errors, however, would not explain why humans fail to abandon problems upon reaching perfect performance.

### Limitations and weaknesses

Several limitations and qualifications must be acknowledged about this work. First, learners did not know that the problems would be unsolvable. This is a departure from the method of Payne and Duggan (2011) who told learners at the outset that some problems would be impossible and thereby observed that learners mostly persisted in problems that were solvable. Unsolvable problems were terminated relatively early on by the learners. This contrasts with findings from the present experiments that learners spent an inordinate number of trials on unsolvable problems. By not making the alternatives known at the outset to the learners, it could be argued that such information would not be entertained as potential hypotheses in the problem space (e.g., Risko et al., 2017), and therefore may explain the different results between the experiments. The expectation of solvability may be exacerbated by the answer-driven nature of education that problems presented in an educational context would have a solution. Reality beyond the laboratory, unfortunately, dictates that not all problems may have solutions. Further, the desired level of success may not be explicitly known to the learner to guide when to stop acquiring information or attempting responses (Ferguson, 1989). Therefore, being an efficient learner in a complex and multifaceted learning space involves decisions involving when to expend resources given unknown outcomes (Chow et al., 1971; Hill, 2009). Educators should be mindful to consider whether they are doing students a disservice by setting the expectation that all problems are solvable.

Second, a plausible argument may be made that the five separate experiments should be amassed into one dataset that would allow more efficient analysis and permit a direct comparison across experiments. Doing so, however, would not accurately reflect the sequential reasoning that occurred as the results of one experiment were used to guide future experiments. One of the key tenets of sound between-group design is independence between conditions, which would be violated by combining experiments.

And finally, concerns may be raised about the representativeness and adequacy of the sample. Indeed, this issue may be leveled at most studies that use college students and convivence sampling. The sample obtained in the present experiments contained a predominance of participants who were Caucasian and female. How this may affect learning cessation in the current experiments is unknown, but it is possible that past experiences in learning and cultural expectations may predispose certain participants to be inclined to cease learning, a point that we raise in the next section on future directions.

### Future directions

Given the unexpected nature of the results, there are several questions that must be explored in future projects regarding reasons for the lack of cessation and its implications for education. First, given the importance of self-control and regulation processes called executive functioning (Diamond, 2002; Davidson et al., 2006), it is possible that the failure of cessation may be related to a deficiency in inhibition. Second, another source of reasons why some individuals cannot cease learning may be related to temperamental or personalistic factors (Evans and Rothbart, 2007; Rothbart et al., 2007) such as having a goal versus performance orientation (Elliott and Dweck, 1988; Wang et al., 2021) and, of course, whether grit plays a role in cessation (Duckworth et al., 2012a, 2012b). Third, there should be some consideration of the influence of the environment and expectations of one’s culture regarding learning (Kruger and Tomasello, 1996). It is plausible that past parenting practices and messages from the schooling culture that emphasize perfection or persistence may bias an individual against abandoning learning attempts rather than focusing on the success or outcome of one’s learning (Corcoran, 2014). And fourth, considering that humans did not conform to the predictions of machine learning models, it would be instructive to use computational modeling to simulate the extended learning performance of humans and thereby elucidate processes and mechanisms behind protracted learning. Simply stated, is it possible (yet obviously not desirable) to make machine learners as inefficient as humans?

Finally, the ability to self-regulate learning including cessation in a manner that is effective, efficient, and sensitive to feedback has implications for educational success that deserve further exploration (Van Loon and Oeri, 2023). Efficient and effective learning would involve managing limited cognitive resources by exploiting information from fruitful learning opportunities but abandoning unproductive attempts. Therefore, it is conceivable that individual differences in learning cessation may relate to performance in other educational outcomes. It is also intriguing to speculate whether attempts to promote deep learning through educational practices in humans may have some bearing on the likelihood to cease learning (Darling-Hammond et al., 2019; Mehta and Fine, 2019; Rickles et al., 2019; Song et al., 2022). Caution is warranted though as the time scale of the present task was microanalytic as cessation was measured on a trial-by-trial basis. Other decisions to quit a learning task may occur at a molar level such as days, months, semesters, or years. It is therefore difficult to extrapolate from quitting in a feedback-based learning task to longer-term learning or broader educational contexts. Answers to these questions though may have some bearing on messages that are sent to students regarding whether persistence, regardless of success, is always the best strategy.

## Data availability statement

The raw data supporting the conclusions of this article will be made available by the authors, without undue reservation.

## Ethics statement

The studies involving human participants were reviewed and approved by YSU Institutional Research Board. The patients/participants provided their written informed consent to participate in this study.

## Author contributions

JC contributed to all phases of this investigation.

## Conflict of interest

The author declares that the research was conducted in the absence of any commercial or financial relationships that could be construed as a potential conflict of interest.

## Publisher’s note

All claims expressed in this article are solely those of the authors and do not necessarily represent those of their affiliated organizations, or those of the publisher, the editors and the reviewers. Any product that may be evaluated in this article, or claim that may be made by its manufacturer, is not guaranteed or endorsed by the publisher.
